# Supplementary material for: Development of a novel eye drop aid and evaluation of its efficacy
Source: PLoS One. 2025 Sep 19;20(9):e0332533. doi: 10.1371/journal.pone.0332533 (PMC12448970; doi:10.1371/journal.pone.0332533)
Supplement: Attachment — Submitted filename: reply letter for reviewer 2.docx [file pone.0332533.s003.docx]

Reviewer #2:

Thank you for your valuable comments, I reply to your comments below. Revised points are highlighted in yellow.

Comment:

Introduction
You may want to add a connective word like 'However,' to the sentence starting with 'Many patients'.
You may want to add a connective word like 'In an effort to resolve this issue' to the sentence starting with Many eyedrop instillation aids.
Reply:

The manuscript has been reviewed by a native English editor to improve overall language quality, including transitions and coherence.

Comment:
You have to add a colon (:) after [12-16].
Reply:

Corrected.
Comment:

Patients
The sentence starting with 'With respected to corrected visual acuity' should be changed to 'With respect to'.
Reply:

Corrected.
Comment:

Discussion
You have to omit " at the third paragraph.
You may want to change 'However' to 'In addition' in the last sentence of the fourth paragraph.
Reply:

Corrected.
Comment:

You have to change 'only an eye drop aid' to 'only an eyedrop aid' to align with the terminology employed in other parts of the manuscript.
You have to omit " at the end of the Discussion.

Corrected.
